# Correction: Computational study of linear carbon chain based organic dyes for dye sensitized solar cells

**DOI:** 10.1039/d4ra90037e

**Published:** 2024-04-11

**Authors:** Giuseppe Consiglio, Adam Gorczyński, Salvatore Petralia, Giuseppe Forte

**Affiliations:** a Department of Chemical Science University of Catania Via S. Sofia 64 95125 Italy; b Faculty of Chemistry, Adam Mickiewicz University Uniwersytetu Poznańskiego 8 61-614 Poznań Poland; c Department of Drug Science and Health University of Catania Via S. Sofia 64 95125 Italy gforte@unict.it

## Abstract

Correction for ‘Computational study of linear carbon chain based organic dyes for dye sensitized solar cells’ by Giuseppe Consiglio *et al.*, *RSC Adv.*, 2023, **13**, 1019–1030. https://doi.org/10.1039/D2RA06767F.

The authors regret that the name of one of the authors (Adam Gorczyński) was shown incorrectly in the original article. The corrected author list is as shown here.

The Royal Society of Chemistry apologises for these errors and any consequent inconvenience to authors and readers.

## Supplementary Material

